# Influence of maternal age, gestational age and fetal gender on expression of immune mediators in amniotic fluid

**DOI:** 10.1186/1756-0500-5-375

**Published:** 2012-07-24

**Authors:** Tobias Weissenbacher, Rüdiger P Laubender, Steven S Witkin, Andrea Gingelmaier, Barbara Schiessl, Franziskus Kainer, Klaus Friese, Udo Jeschke, Darius Dian, Katrin Karl

**Affiliations:** 1 Department of Obstetrics and Gynaecology, Ludwig-Maximilians-University, Maistrasse 11, Munich, 80337, Germany; 2 Department of Obstetrics and Gynaecology, Weill Medical College of Cornell University, New York, USA; 3 Department of Obstetrics and Gynecology, University Hospital, Aachen, Germany; 4 Institute of Medical Informatics, Biometry and Epidemiology, Ludwig-Maximilian-University, Munich, Germany

**Keywords:** Amniotic fluid, Gestational age, Fetal gender, Cytokines, Immune mediators

## Abstract

**Background:**

Variations in cytokine and immune mediator expression patterns in amniotic fluid due to gestational age, maternal age and fetal gender were investigated.

**Findings:**

Amniotic fluid samples were obtained from 192 women, 82 with a mid-trimester amniocentesis (median gestational age 17 weeks) and 110 with a caesarean section not in labor (median gestational age 39 weeks). Amniotic fluid was screened by commercial ELISAs for the TH1/TH2/TH17 cytokines and immune mediators IL-1 beta, IL-2, IL-4, IL-6, IL-8, IL-10, IL-12, IL-15, IL-17, TNF alpha, GRO-alpha, MIP1alpha, MIP1beta, Histone, and IP10. Analysis was by Bonferroni correction for multiple comparisons. None of the 15 examined cytokines revealed any differences in expression patterns regarding fetal gender. Significant differences were found in IL-4, IL-10, IL-12, TNF- alpha, GRO-alpha and MIP1-beta with respect to gestational age and in GRO-alpha regarding maternal age.

**Conclusion:**

Cytokines utilized as biomarkers in the diagnosis of intrauterine infections are not influenced in their expression pattern by fetal gender but may vary with respect to maternal age and gestational age.

## Background

The diagnosis early in gestation of pregnancy complications such as infection/inflammation, preeclampsia and intrauterine growth restriction would allow the maximum time for initiation of treatment and monitoring. Clinical manifestations of disease often appear too late to allow for meaningful intervention. Commonly used parameters, such as measurement of C-reactive protein (CRP), leukocyte subsets and concentrations of pro-calcitonin are nonspecific and therefore unreliable [1,2]. In addition, obese women demonstrate higher levels of CRP and tumor necrosis factor (TNF)-alpha when compared to patients with lower body mass index (BMI) [3]. Furthermore, chorioamnionitis (CAM) is a histological diagnosis obtained after delivery and, therefore, is not a diagnostic tool.

Many studies have investigated utilizing amniotic fluid samples to detect differences in analyte concentrations as biomarkers for pathological gestations [4,5]. The findings to date remain inconclusive. The measurement of interleukin 6 (IL-6) in the amniotic fluid (AF) is currently a frequently reported method to diagnose an intrauterine infection [6-8]. Other cytokines, especially IL-8, have also been suggested to have value in the diagnosis of an intrauterine infection [6,9]. Recent studies indicate that inter-individual differences in cytokine levels might be due to numerous factors including BMI [3], intrauterine growth restriction, parity [10] or maternal or fetal pathologies. Elevated levels of IL-6, IL-8 and IL-10 were described in patients with hyperechogenic bowel [4] and reduced cytokine levels in amniotic fluid were reported in patients with Down`s syndrome fetuses [5]. Furthermore, elevated IL-10 levels in amniotic fluid were detected in small for gestational age pregnancies [10]. Ethnicity is another potential influencing factor on cytokine levels [11]. Therefore, intraamniotic cytokine values may not always be suitable as markers for intrauterine infection [12].

A shortcoming of many amniotic fluid immune mediator studies is the paucity, absence or consistency of data on intraamniotic variations with gestational or maternal age or with fetal gender. Few cytokines have been investigated with respect to differences in gestational age. Levels of IL-6, IL-1beta and tumor necrosis factor alpha (TNF-alpha) have been reported in some investigations to increase throughout gestation and are highest at term [13,14]. Other studies could not detect TNF-alpha and IL-1beta throughout normal pregnancies and reported constant levels of IL-6 and IL-8 [15]. Investigations of expression patterns of additional potential immune biomarkers in amniotic fluid with respect to gestational age, as well as studies relating immune mediator concentration to the age of the mother and sex of the fetus, have not been reported.

In the present study, we investigated concentrations of 15 immune mediators [IL-2, IL-17, IL-4, IL-6, IL-8, IL-10, IL-12, IL-15, growth regulated oncogen-alpha (GRO-alpha), macrophage inflammatory protein (MIP1alpha), MIP1beta, Histone, TNF -alpha, IL1beta, IP10] of the T-helper (TH) TH1, TH2 and TH17 pathway in human amniotic fluid with respect to gestational age, gender of the fetus and age of the mother. Knowledge of baseline concentrations of immune mediators in amniotic fluid throughout pregnancy would improve our ability to analyze variations in levels as a diagnostic tool.

## Methods

### Study design and population

Subjects were attending consultations in the Department of Obstetrics and Gynecology in the University Hospital, Munich between 2007 and 2010. All patients were interviewed regarding complaints, antibiotic use and history of sexually transmitted diseases and vaginal infections. Patients were screened for abnormalities in fetal development at the enrolment visit. The institutional review board of the Ludwig-Maximilians-University Munich, Germany, approved this study and all patients gave written informed consent prior to collection of amniotic fluid.

A total of 192 patients were included in this study and subdivided into two groups: 1) Women at term not in labor who received caesarean section (CS) [n = 110, median gestational age (mga) = 39 weeks], 2) patients who received an amniocentesis (AC) because of advanced maternal age, patient’s request or because of an increased risk for aneuploidies due to sonographic aberrations in nuchal translucency or abnormal levels of serum biochemical markers [n = 82, mga = 17 weeks]. All patients delivered in our hospital and gave birth to healthy infants with no signs of infection or abnormality. The number of patients, range of gestational age as well as the median gestational weeks are listed in Table 1.

**Table 1:** Gestational weeks and neonatal outcome of the examined groups with case numbers

**Neonatal outcome**	**n**	**Amniocentesis**	**Caesarian section**
Gestational week	186	17 (15–31)	39 (30–42)
Birth weight in gram	135	3340 (1480–4060)	3170 (1210–4668)
Birth-pH	130	7.32 (7.10-7.50)	7.33 (7.13-7.44)
Birth-base excess	127	−3.0 (−10.2-2.0)	−2.0 (−21.0-1.2)
APGAR min 1	135	9 (0–10)	9 (2–10)
APGAR min 5	135	10 (8–10)	10 (6–10)
APGAR min 10	135	10 (8–10)	10 (8–10)

Median and range (in brackets) for continuous variables, percentages and number of patients (in brackets) for discrete variables.

### Sample collection

None of the patients who received a caesarean section had a membrane rupture prior to surgery and sample collection. Amniotic fluid was obtained during caesarean section following uterotomy and amniotomy. Five ml amniotic fluid were obtained with a sterile syringe. In women undergoing amniocentesis, samples were obtained transabdominally. As a standard procedure, ultrasound was initially performed to evaluate the position of the fetus, the amount of amniotic fluid and the site of the placenta. A 20 G x 3.5 needle was used and guided under sonographic monitoring into the amniotic cavity. Amniotic fluid was first taken for further diagnostic tests dependant on the indication of the invasive procedure. Afterwards, 5 ml amniotic fluid were removed with a syringe and further processed as specified below. Samples were transported immediately in a capped sterile syringe to the laboratory, divided into 1 ml aliquots and stored at −80 °C until analyzed. Samples were stored for four month in average until measurement.

### Measurement of cytokines

Immune mediators in the amniotic fluid were measured utilizing commercially available enzyme linked immunosorbent assay (ELISA) kits (R&D Systems, Minneapolis, MN, USA). A standard curve was obtained in parallel to each assay and results converted to pg/ml.

### Statistical analysis

Data were summarized by adequate measures of location and spread for continuous variables and by proportions for discrete variables. Differences in the cytokines between gestational age (Table 2) and fetal gender (Table 3) were each evaluated by Mann–Whitney U tests. Differences in cytokine expression with respect to the age of the mother (Table 4) were assessed by the Kruskal-Wallis test. Furthermore, we modelled the differences in the cytokines between AC and CS adjusted for maternal age and sex of the fetus by using linear regressions. By analogy, we modelled the differences in the cytokines between week of gestation adjusted for maternal age and sex of the fetus. For each regression model we used complete cases. In order to satisfy the normality assumption of the linear regression models, we transformed each of the cytokines by taking the natural logarithm. Graphically based residual analyses were performed for checking the assumptions of each of the fitted linear regression models. The impact of the independent variables (AC vs. CS, gestational age, fetal sex, age of the mother) on the cytokines is quantified by corresponding regression coefficients β. Each regression coefficient is tested by using the t-test (two-sided) with n–k degrees of freedom where n denotes the sample size and k the number of regression coefficients in the regression model. As many cytokines were tested and modelled we had to correct the p-values for multiple testing. For that purpose we used the procedure of Bonferroni-Holm. However, for conventional purposes we report the uncorrected p-values and compare them to the corrected ones. Hence, a corrected p-value of <0.05 was considered as statistically significant. All statistical analyses were performed by using R (version 2.12.2) and SPSS (version 16.0).

**Table 2:** Indications for caesarian section (with total number and percentage)

**Indication for C.section**	**n**	**percentage**
C.section in history	43	39.1%
Breech presentation	19	17.3%
Multiples	8	7.3%
Uterus surgery in history	5	4.5%
Whish of the patient	4	3.6%
Others	31	28.2%

**Table 3:** Cytokine and immune mediator concentrations (pg/ml) in amniotic fluid obtained during the second and third trimester – univariate analysis

**Mediator**	**Median concentration in pg/ml (range) Second trimester**	**Median concentration in pg/ml (range) Third trimester**	**p-value**
IL-2 (n = 66)	2.1 (1.8-4.6)	4.5 (0.8-9.1)	0.054
IL-4 (n = 182)	10.0 (2.6-12.3)	7.1 (0.8-21.9)	0.022
IL-6 (n = 156)	1141.5 (91.2-2823)	764.2 (120.8-21275.0)	0.953
IL-8 (n = 185)	1035.1 (235.7-4295.8)	628.8 (0–17125.0)	0.111
IL-10 (n = 161)	4.9 (2.2-26.6)	6.6 (2.4-85.7)	0.037
IL-12 (n = 161)	4.8 (2.7-10.9)	6.8 (2.0-23.8)	0.038
IL-15 (n = 173)	15.4 (2.4-32.9)	35.0 (2.6-169.5)	0.109
IL-17 (n = 66)	17.5 (4.6-42.4)	23.1 (3.9-46.0)	0.645
TNF- α (n = 161)	22.1 (2.7-68.7)	10.5 (4.8-659.0)	0.001
GRO- α (n = 184)	248.8 (11.6-7029.7)	1252.3 (21.3-1658.8)	0.003
Pro IL1-ß (n = 157)	20.3 (15.2-43.2)	16.5 (0–8048.8)	0.16
Histone (n = 161)	0.15 (0.05-0.55)	0.29 (0.04-78.39)	0.156
IP-10 (n = 169)	1042.5 (574.4-2696.1)	681.3 (5.4-5184.9)	0.100
MIP1-ß (n = 182)	116.1 (16.1-485.2)	25.9 (5.9-16305.8)	0.001
MIP1- α (n = 186)	23.2 (13.8-153.2)	26.2 (0–1531.3)	0.818

**Table 4:** Cytokine and immune mediator concentrations (range and median concentration in pg/ml) in amniotic fluid by fetal gender

**Mediator**	**Concentration range (median) male**	**Concentration range (median) female**	**p-value**
IL-2 (n = 66)	0.4-7.5 (4.7)	0.8-9.1 (3.9)	0.537
IL-4 (n = 182)	1.2-21.5 (8.6)	0.8-21.9 (8.3)	0.600
IL-6 (n = 156)	237.3-21275.0 (904.2)	120.8 -15825 (662.0)	0.079
IL-8 (n = 185)	0-5362.5 (664.9)	0-17125 (600.8)	0.644
IL-10 (n = 161)	2.4-18.5 (5.8)	2.2-85.6 (6.5)	0.559
IL-12 (n = 161)	0.7-23.7 (5.6)	0-17.3 (6.1)	0.478
IL-15 (n = 173)	2.5-169.4 (16.3)	2.4-165.8 (27.7)	0.868
IL-17 (n = 66)	3.5-47.9 (23.1)	4.1-39.7 (23.2)	0.940
TNF- α (n = 161)	5.3-30.3 (11.3)	2.9-659 (11.1)	0.870
GRO- α (n = 184)	14.5-7658.8 (1021.9)	12.3-6106 (1079.9)	0.985
Pro IL1-ß (n = 157)	0-269.1 (16.9)	2-1048.8 (18.1)	0.797
Histone (n = 161)	0.04-3.7 (0.2)	0.06-78.4 (0.3)	0.654
IP-10 (n = 169)	11.6-6781.6 (678.3)	0.4-32370 (703.6)	0.914
MIP1-ß (n = 182)	10.5-188.3 (25.4)	5.8-16305.8 (25.4)	0.590
MIP1- α (n = 186)	7.9-86.3 (26.1)	0-1531.3 (25.4)	0.410

## Results

192 women were screened for cytokine concentrations, 110 from those who underwent a CS and 82 who had an amniocentesis. Of the CS subjects, 104 received a primary caesarean section while 6 underwent a secondary caesarean section due to fetal distress. None of these six cases had rupture of membranes.

The number of cases differed within each cytokine due to missing completed at random, causally because of some cases being out of measuring range.

### Gestational age

The gestational age in the CS-group ranged from 30 to 42 weeks (mga 39). Within the AC-group the gestational age ranged between 15 and 31 weeks (mga 17.6) (Table 1). Moreover, neonatal outcome with birth weights, APGAR-scores, birth-pH and base excess is demonstrated in Table 1. Table 2 demonstrates the indications for C. section.

No statistically significant difference was found between the AC and CS groups regarding cytokine levels of IL-2, IL-17, IL-6, IL-8, IL-15, MIP1 alpha, Histone, GRO-alpha, ProIL-1beta or IP10. In the univariate analysis, IL-4, TNF-alpha, GRO-alpha and MIP1-beta demonstrated significantly elevated levels in the second trimester (AC-group) compared to the third trimester (CS-group) with p = 0.022, p = 0.001, p = 0.003 and p = 0.001 respectively (Table 3). IL-10 and IL-12-levels were significantly lower in the second trimester when comparing to the third trimester (p = 0.037 and p = 0.038) (Table3).

### Gender of the fetus

82 samples were collected in the amniocentesis group. We found a distribution of 31% boys and 69% girls. In the group with caesarean section, 59 boys (53.6%) and 51 girls (46.4%) were evaluated. Univariate analysis revealed no statistically significant differences between fetal gender with regard to expression of any of the cytokines (Table 4).

### Maternal age

Maternal age ranged from 19.3 to 48.6 years in the total sample. In the amniocentesis group the range was 19.3 to 45.6 years (median age 36.5 years); in the group with caesarean section maternal age ranged from 22.5 to 48.5 years (median age 34.47 years). GRO- alpha demonstrated higher levels in patients who were 26–30 years and 31–35 years compared to the other age groups (p = 0.01). None of the other 14 examined cytokines and chemokines revealed any differences in expression patterns with regard to maternal age (Table 5).

**Table 5:** Cytokine and immune mediator concentrations in amniotic fluid by maternal age

**Mediator**	**Age**	**Age**	**Age**	**Age**	**Age**	**p-value**
	**19-25**	**26-30**	**31-35**	**36-40**	**>40**	
IL-2 (n = 66)	5.2	4.6	4.3	3.8	3.9	0.97
IL-4 (n = 182)	8.3	6.6	9.0	9.2	9.7	0.18
IL-6 (n = 156)	879.2	668.9	738.7	669.2	832.8	0.97
IL-8 (n = 185)	829.5	551.4	754	621.0	552.1	0.45
IL-10 (n = 161)	9.1	7.1	5.8	5.5	5.1	0.49
IL-12 (n = 161)	5.1	5.8	4.9	5.3	5.5	0.94
IL-15 (n = 173)	35.8	31.3	28.0	12.3	11.5	0.51
IL-17 (n = 66)	32.7	19.3	19.2	23.4	21.8	0.46
TNF- α (n = 161)	9.3	11.0	12.1	13.0	12.1	0.56
GRO- α (n = 184)	629.5	1254.3	1228.1	663.0	492.9	0.01
Pro IL1-ß (n = 157)	19.8	19.5	16.9	19.4	17.0	0.48
Histone (n = 161)	0.3	0.2	0.3	0.3	0.3	0.83
IP-10 (n = 169)	1435.3	644.6	722.7	814.0	702.8	0.33
MIP1-ß (n = 182)	27.5	25.8	28.3	25.9	27.7	0.71
MIP1- α (n = 186)	25.6	25.9	25.7	24.8	26.0	0.79

Median biomarker concentration (pg/ml) by maternal age.

## Discussion

The identification of specific immune mediators and their concentration in amniotic fluid is receiving increasingly attention as potential aids for making informed diagnostic and therapeutic decisions. This field remains unsettled, however, due to a lack of consistency between observations in different investigations as well as a scarcity of longitudinal data.

The composition of amniotic fluid changes during pregnancy which was investigated by Unerwood et al [16]. Accordingly, AF changes its milieu during pregnancy and is influenced by various factors such as gestational week, uterine perfusion and many more. Especially in the second half of pregnancy AF composition seems to change [17].

Factors influencing cytokine expression patterns were examined in the past and numerous variables such as ethnicity, fetal diseases, small for gestational age pregnancies and body mass index were described [4,5,11]. Especially differences in cytokine expression patterns regarding ethnicity were already described in literature [11,18-21] and available data seem to be more consistent when comparing to available data regarding fetal gender or gestational week. Data are missing concerning varieties of cytokine levels due to gestational week, fetal gender or mothers` age and only few cytokines have been reported with respect to these factors recently.

Bamberg et al investigated IL-6, IL-8 and TNF-alpha in midtrimester amniotic fluid in pregnancies with normal outcome. The cytokine concentrations in this study were correlated with gestational week, fetal gender and parity [22]. In this study AC was performed between 15 + 0 and 20 + 6 weeks of gestation. Accordingly, no correlation of cytokine levels with parity or fetal gender could be found which underlines our results in these three cytokines. Moreover, we found also no correlation between fetal gender and mothers` age when looking at the cytokines IL-1 beta, IL-2, IL-4, IL-12, IL-15, IL-17, MIP1alpha, MIP1beta, Histone, and IP10. Regarding gestational week, the authors found TNF-alpha values differing between the 15^th^ and 16^th^ and the 15^th^ and 18^th^ week.

Our results concerning TNF-alpha are consistent as we demonstrated elevated TNF-alpha levels related to gestational age. We investigated however further twelve cytokines and demonstrated also elevated IL-4, TNF-alpha, GRO-alpha and MIP 1-beta levels and lower IL-10 and IL-12 levels with regard to gestational week. Consistent data that investigated cytokine levels in amniotic fluid throughout pregnancy cannot be found in the literature.

Chow et al. investigated differences in cytokine expression according to maternal age and fetal gender. IL-1ra levels were demonstrated to increase with maternal age. The fetal gender had an influence on IL-5 levels which were higher in amniotic fluid of male fetuses [23]. We did not look for IL-5 and IL-1ra in our study and therefore were not able to compare our results on this point. However, the cytokines IL-12, IL-15, IL-17, MIP-alpha, MIP-beta and TNF-alpha were investigated in Chow`s and in our study and no differences were found in respect to maternal age and fetal gender which underlines our findings concerning these cytokines.

Contrasting, Bry et al found IL-1ra correlating to fetal gender with higher amniotic fluid concentrations in female fetuses. Length of gestation did not affect the IL-1ra levels [24]. Similar results were published by Romero with a significant effect of fetal gender related to IL-1ra levels [25]. As opposed to this IL-6 and IL-10 was investigated in midtrimester amniotic fluid by Poggi et al. and the authors found fetal gender not affecting the IL-6 and IL-10 levels in amniotic fluid. However the authors additionally investigated angiogenin and demonstrated lower angiogenin levels in amniotic fluid of male fetuses [26]. So according to the available literature data remain inconsistent.

IL-5 and IL-1ra seem to be influenced by fetal gender, whereas other cytokines are unaffected. Compared to other studies, we investigated cytokines that were not investigated so far relating to fetal gender, gestational week and mothers’ age. At least IL-6, IL-8, IL-10 and TNF-alpha that were already described in the literature seem not to be affected by fetal gender, which we could underline and differing to previous studies demonstrate IL-1 beta, IL-2, IL-4, IL-12, IL-15, IL-17, GROalpha, MIP1alpha, MIP1beta, Histone, and IP10 also not being influenced by fetal gender.

A lack of consistency also exists between studies that investigate individual cytokine expression with respect to gestational age.

IL-2 was found to be different between 16^th^ – 18^th^ week and term pregnancies [27]. In other studies IL-1, IL-2, IL-6 and TNF-alpha seem to increase towards term [28][13]. The gestational age was not equally distributed in these studies as well as in our study while the early third trimester weeks are underrepresented. As regular amniocentesis represents an invasive procedure during pregnancy, it is most likely that is would not be approved for research by any ethical committee. Therefore the detection of cytokine levels dependant on gestational age is not investigated prospectively and consistently so far. However, we investigated within a wide range of gestational age (15^th^ – 41^th^ week) and found elevated IL-4, TNF- alpha, GRO-alpha and MIP1-beta levels and decreased levels of IL-10 and IL-12 in the second trimester compared to the third trimester.

Data influencing cytokine expression by mothers’ age, fetal gender and gestational age are inconsistent and longitudinal data are missing. When looking at published studies, especially IL-1ra seems to be influenced by fetal gender.

## Conclusions

In our study none of the investigated cytokines were influenced by fetal gender or mothers’ age. It appears obvious, however, that some cytokines in amniotic fluid vary in concentration by gestational week. This needs to be taken into account, when using these biomarkers as a diagnostic tool. Furthermore, gestational age should be considered when interpreting cytokine levels in amniotic fluid and evaluating the clinical utility of measuring amniotic fluid cytokines.

## Abbreviations

AC: Amniocentesis; AF: Amniotic fluid; BMI: Body mass index; CAM: Chorioamnionitis; CRP: C-reaktive protein; CS: Caesarean section; ELISA: Enzyme-linked immunosorbent assay; GRO: Growth regulated oncogen; IL: Interleukin; mga: Median gestational age; min: Minute; MIP: Macrophage inflammatory protein; TH: T-helper; TNF-α: Tumor necrosis factor alpha.

## Competing interests

The authors declare that they have no competing interests.

## Authors’ contributions

TW designed the study and performed collection, analysis and interpretation of data and drafted the manuscript for publication. RL performed the statistical analysis. SW participated in the design of the study, in the analysis and interpretation of the data and approved the English. AG, BS and FK performed participant inclusion, collected samples and contributed substantially to acquisition of data. FK participated in the design of the study, dada analysis and data interpretation. UJ, DD and KK helped substantially to draft the manuscript. All conceived of the study, participated in its design and coordination, helped with data interpretation and drafting of the manuscript. All authors read and approved the final manuscript.
